# Socioeconomic and Demographic Factors Associated With Mortality Before and During the COVID-19 Pandemic: An Analysis of 28 European Countries

**DOI:** 10.3389/ijph.2025.1608560

**Published:** 2025-07-23

**Authors:** Paola Sillitti, Clément Meier, Olivier Mucchiut, Jürgen Maurer, Ralf J. Jox

**Affiliations:** ^1^ Faculty of Business and Economics (HEC), University of Lausanne, Lausanne, Switzerland; ^2^ Swiss Centre of Expertise in the Social Sciences (FORS), University of Lausanne, Lausanne, Switzerland; ^3^ Swiss Center of Expertise in the Life Course Research (LIVES), Lausanne, Switzerland; ^4^ Lausanne University Hospital (CHUV), Lausanne, Switzerland; ^5^ Institute of Humanities in Medicine, Lausanne, Switzerland

**Keywords:** end of life, COVID-19, pandemic, socioeconomic inequalities, health inequalities

## Abstract

**Objectives:**

The pandemic was the most significant event affecting health systems in the last 100 years. Research shows that gender, age and socioeconomic status were associated with higher mortality during the pandemic. However, most studies are cross-sectional and country specific. This paper assesses sociodemographic characteristics associated with time and cause of death in Europe between 2018 and 2022.

**Methods:**

The analysis includes 7,137 decedents aged over 50, using post-death interviews with proxy respondents, from the Survey on Health, Ageing, and Retirement in Europe (SHARE). Data from 28 countries, from SHARE waves 7 to 9, are examined using t-tests, chisquaretests and multivariate logit regression models, controlling for sociodemographic characteristics. The three binary outcome variable sindicate the time and cause of death.

**Results:**

Being male, older, without a partner, self-reporting financial difficulties, and living in Eastern Europe were associated with an increased likelihood of dying during the pandemic. The association was stronger for deaths due to COVID-19, respiratory and infectious diseases.

**Conclusion:**

The pandemic highlighted socioeconomic gradients in mortality. These results call for policymakers to prepare for future shocks, ensuring equal access to adequate care.

## Introduction

The COVID-19 pandemic has been the most significant event affecting health systems in the last 100 years [1, 2]. As of September 2024, over 776 million cases of COVID-19 were recorded globally, and over 7 million people died due to COVID-19, with such data likely underestimating the infections and deaths due to undetected case [3]. Furthermore, while the World Health Organisation (WHO) declared the end of the public health emergency of international concern in May 2023, COVID-19 did not cease to impact people’s lives and health systems [4]. Governments across the world supported health systems by implementing emergency policies during the early stages of the pandemic, such as creating task forces and rapid response teams, increasing critical care beds, and implementing short-term policies to increase and reward healthcare staff to limit staff shortages [5]. Although many countries adopted emergency policies to mitigate the impact of the COVID-19 pandemic, the effectiveness of these measures varied widely. In the early phase, some countries faced critical shortages of protective equipment such as masks and hand sanitiser, which hampered efforts to slow the spread of the virus [6]. Coming out of the public health emergency, governments across the world must improve the resilience of their health systems to future shocks [2]. Evidence is needed today to understand what factors were associated with mortality during the COVID-19 pandemic, to guide an evidence-based approach to policymaking [2].

Socioeconomic gradients in health and healthcare are not new. A solid batch of literature has already largely studied the socioeconomic inequalities and inequities in health status and healthcare access before the pandemic [7–10]. However, the shock caused by an unexpected crisis like the COVID-19 pandemic represented an opportunity to study socioeconomic gradients of mortality and inequalities and inequities in health and healthcare access in times of crisis. Previous literature has indicated that people of older age [6, 11–13], men [11, 12, 14, 15], and ethnic minorities [11, 12, 14–20] were disproportionately impacted by the pandemic and had a higher likelihood of dying from COVID-19.

Furthermore, studies analysing the contagion of COVID-19 showed that at the onset of the pandemic people with higher socioeconomic status were more exposed to COVID-19 due to more frequent travelling, while people with lower socioeconomic status were more exposed to the disease in the subsequent phases of the pandemic, once measures of social distancing were implemented [14, 17, 21–23].

Looking at the excess mortality and mortality rates due to COVID-19, a number of studies have shown that people with lower socioeconomic status and/or living in more deprived areas were more likely to die during the COVID-19 pandemic. In England, France, Italy and the United States, areas with lower socioeconomic status showed higher levels of hospitalizations and mortality during the pandemic [12, 21, 23–25]. In Germany, the effect of the pandemic on mortality and hospitalisations was greatest in counties with low education and least pronounced in counties with high education [26]. Individuals with lower income and lower socioeconomic status were found to be more likely to die during the pandemic in Belgium and Sweden [27–29].

While the existing literature indicates that individuals’ sociodemographic characteristics may have been associated with higher mortality during the COVID-19 pandemic, most results come from cross-sectional country-specific studies using national data. To our knowledge, no previous studies have explored the sociodemographic characteristics associated with mortality during the pandemic using a comprehensive longitudinal dataset across multiple countries.

Taking stock of the characteristics of people who died before and after the onset of the pandemic, this paper aims to contribute to a better understanding of what demographic and socioeconomic characteristics of decedents are associated with the moment and cause of death. Thus, using longitudinal microdata from the Survey on Health, Ageing, and Retirement in Europe (SHARE), this paper addresses the gap in the literature by examining the demographic and socioeconomic factors associated with mortality among older adults in Europe before and after the onset of the pandemic. It also compares the socioeconomic characteristics of individuals for whom COVID-19 was the leading cause of death with those who died from other causes.

The Survey of Health, Ageing and Retirement in Europe is a multidisciplinary and cross-national database of microdata collected through interviews to individuals aged 50 or older from 28 European countries. These interviews gather detailed information about demographic and socioeconomic characteristics of older Europeans, their health status and healthcare use, as well as their social networks. This approach provides valuable insights into the characteristics of older Europeans, including their end-of-life experiences.

## Methods

### Study Design and Participants

The analysis includes microdata of 7,137 decedents aged above 50 years, collected through interviews with proxies of survey participants after the respondent’s death, from SHARE [30–33]. These end-of-life interviews collect information on the decedent’s sociodemographic characteristics, health status, care received, experiences with health and care in the last year before death, and the circumstances of death. The analysis uses pooled data from waves 7 to 9 (2018–2022) of SHARE collected in 28 countries (Supplementary Table SA.1). The analysis focuses on people whose death occurred from 2 years before until 2 years after the outbreak of the pandemic. Such a restriction in the sample allowed for better comparability of the sociodemographic characteristics of the decedents whose deaths occurred before and after the outbreak of the pandemic. Sensitivity analyses restricted to deaths occurring after March 2020 and to COVID-19–related deaths only were performed and yielded substantively similar results. For parsimony and statistical power, we report models based on the full 2018–2022 sample.

### Outcome Variables

#### Time of Death

This outcome variable describes whether the deceased died before or after the outbreak of the pandemic, in March 2020. The variable takes the value 0 if the deceased died before March 2020 (In March 2020, the WHO declared the COVID-19 outbreak a global pandemic [34]) and value of 1 otherwise. It is built on two questions asked to the proxy respondent of the deceased person (Supplementary Table SA.2).

#### Cause of Death (COVID-19)

The second outcome variable summarises the cause of death of the deceased. The variable has a value 1 if COVID-19 was the leading cause of death and value 0 if the deceased died from other causes. It is built on one question of the SHARE end-of-life interview (Supplementary Table SA.2).

#### Cause of Death (Respiratory and Infectious Diseases, Including COVID-19)

It is possible that, particularly in the early phases of the pandemic, deaths happening due to COVID-19 were underreported due to misinterpretation of symptoms or lack of guidelines for the certification and classification of COVID-19 as a cause of death [35–37]. To include all deaths due to COVID-19 in our analysis, we built a third outcome variable, which takes value 1 if the leading cause of death was reported to be a respiratory disease, an infectious disease (e.g., pneumonia and flu), or COVID-19, and value 0 if the deceased died from other causes. It is built on one question of the SHARE end-of-life interview (Supplementary Table SA.2).

### Covariates

The analysis considers key demographic and socio-economic characteristics of the decedents, such as sex, age, health status, partnership status, education level, financial difficulties, and geographical area. Sex is categorized as male or female. Age is categorized into three age groups (50–74, 75–84, 85+ years). Health status is classified as poor/fair health, good health or very good/excellent health, based on self-reported information that the decedent had provided in the last interview before death. Partnership status is categorized as whether the deceased had a partner or not. Education level is classified into three groups, based on the International Standard Classification of Education (ISCED). The three groups correspond to low (corresponding to ISCED levels 0-1-2), middle (ISCED levels 3-4) and high education (ISCED levels 5-6) [38]. The variable on financial difficulties is self-reported and is classified as whether the deceased had reported their household to be able to make ends meet, in the last wave before death. The variable is categorized as making ends meet easily, fairly easily, with difficulty or with great difficulty. Finally, the variable on the geographical area is categorized as Eastern Europe, Northern Europe, Southern Europe, Western Europe, based on the countries’ classification used in the United Nations’ statistics [39].

### Statistical Analysis

First, the study analyses the bivariate association between each outcome variable and the sociodemographic characteristics of the deceased. It performs a t-test for mean differences and chi-square tests for categorical variables across decedents whose deaths occurred before March 2020 and those whose deaths occurred in March 2020 or later, a t-test and chi-square test across decedents who died due to COVID-19 and those who died for other causes, and a t-test and chi-square test across decedents who died due to respiratory or infectious diseases (including COVID-19) and those who died from other causes. Then, the analysis uses logit models to analyse the association between each outcome variable and the sociodemographic factors, such as sex, age, health status, partnership status, education level, financial difficulties, and geographical area. Due to the SHARE survey design, decedents who died before March 2020 are, on average, 8 months younger than those who died after March 2020. To account for this, Supplementary Tables SB.1, SB.2 include an analysis of the association between each outcome variable and the sociodemographic factors, with stratification by age group (decedents at age 50–79 and decedents at age 80 or older). All statistical analyses were performed using STATA (version 18.0) software. Two-sided p-values <0.05 were considered statistically significant with outcomes presented as average marginal effects (AME) and the associated standard errors (SE). This study is reported in accordance with the STROBE (Strengthening the Reporting of Observational Studies in Epidemiology) guidelines.

## Results

Tables 1–3 show the demographic and socioeconomic characteristics of the sample, respectively by time of death and cause of death, codified as death due to COVID-19 versus death due to other causes in Table 2 and as death due to COVID-19 versus death due to other causes in Table 3. The tables also report the results of the t-test and chi-square test. The categorisation of causes of death is based on predefined response options available in the SHARE end-of-life interview, which includes a limited set of ten broad categories reported by proxy respondents (see Supplementary Table SA.2). For analytical clarity and due to sample size constraints, the variable has been constructed by grouping the causes of death into four main categories, corresponding to cancer, cardiovascular disease, COVID-19 and other. Deaths categorised as cardiovascular diseases include heart attack, strokes and other cardiovascular related illness such as heart failure, arrhythmia. The category of other diseases includes respiratory diseases, diseases of the digestive system such as gastrointestinal ulcer, inflammatory bowel disease, severe infectious diseases such as pneumonia, septicaemia or flu, accident or suicide. A different grouping is used for the last model in the regression analysis, where the category COVID-19 includes respiratory diseases and infectious diseases such as pneumonia, septicaemia or flu.

**TABLE 1:** Sociodemographic characteristics of the study population, by time of death, decedents aged 50+, N = 7,137 (28 European countries. 2018–2022).

Variables	Deceased before March 2020 n (%)	Deceased in March 2020 or later n (%)	T-test/Chi-square test
N	3,545 (49.7%)	3,592 (50.3%)	
Gender			
Male	1,912 (53.9%)	2,005 (55.8%)	0.110
Female	1,633 (46.1%)	1,587 (44.2%)	
Age at death	79.86 years	80.53 years	0.004
Self-rated health (in the last interview before death)			
Poor/fair	2,552 (72.0%)	2,630 (73.2%)	0.299
Good	743 (21.0%)	739 (20.6%)	
Very good/excellent	250 (7.1%)	223 (6.2%)	
Partnership status			
Has a partner	2,138 (60.3%)	2,084 (58.0%)	0.049
No partner	1,407 (39.7%)	1,508 (42.0%)	
Main cause of death			
Cancer	870 (24.5%)	713 (19.8%)	<0.001
Cardiovascular	1,485 (41.9%)	1,362 (37.9%)	
COVID-19	17 (0.5%)	489 (13.6%)	
Other	1,173 (33.1%)	1,028 (28.6%)	
Education level			
Low	1,880 (53.0%)	1,870 (52.1%)	0.705
Medium	1,196 (33.7%)	1,233 (34.3%)	
High	469 (13.2%)	489 (13.6%)	
Ability to make ends meet			
Easily	806 (22.7%)	734 (20.4%)	0.104
Fairly easily	935 (26.4%)	998 (27.8%)	
With difficulty	1,160 (32.7%)	1,183 (32.9%)	
With great difficulty	644 (18.2%)	677 (18.8%)	
Geographical area			
Eastern Europe	824 (23.2%)	974 (27.1%)	<0.001
Northern Europe	483 (13.6%)	474 (13.2%)	
Southern Europe	1,524 (43.0%)	1,508 (42.0%)	
Western Europe	714 (20.1%)	636 (17.7%)	

The table shows the socioeconomic characteristics of the sample, by time of death (before March 2020 vs. in March 2020 or later). The last column reports p-values from the t-test and the chi-square test.

**TABLE 2:** Sociodemographic characteristics of the study population, by cause of death (COVID-19 vs. other causes), decedents aged 50+, N = 7,137, (28 European countries. 2018–2022).

Variables	Deceased due to COVID-19 (%)	Deceased due to other causes (%)	T-test/Chi-square test
N	506 (7.1%)	6,631 (92.9%)	
Gender			
Male	272 (53.8%)	3,645 (55.0%)	0.597
Female	234 (46.2%)	2,986 (45.0%)	
Age at death	80.549 years	80.080 years	0.299
Self-rated health (in the last interview before death)			
Poor/fair	341 (67.4%)	4,841 (73.0%)	0.006
Good	133 (26.3%)	1,349 (20.3%)	
Very good/excellent	32 (6.3%)	441 (6.7%)	
Partnership status			
Has a partner	280 (55.3%)	3,942 (59.4%)	0.070
No partner	226 (44.7%)	2,689 (40.6%)	
Education level			
Low	275 (54.3%)	3,475 (52.4%)	0.267
Medium	175 (34.6%)	2,254 (34.0%)	
High	56 (11.1%)	902 (13.6%)	
Ability to make ends meet			
Easily	74 (14.6%)	1,466 (22.1%)	<0.001
Fairly easily	130 (25.7%)	1,803 (27.2%)	
With difficulty	185 (36.6%)	2,158 (32.5%)	
With great difficulty	117 (23.1%)	1,204 (18.2%)	
Geographical area			
Eastern Europe	209 (41.3%)	1,589 (24.0%)	<0.001
Northern Europe	29 (5.7%)	928 (14.0%)	
Southern Europe	207 (40.9%)	2,825 (42.6%)	
Western Europe	61 (12.1%)	1,289 (19.4%)	

The table shows the socioeconomic characteristics of the sample, by cause of death (COVID-19, vs. other causes). The last column reports p-values from the t-test and chi-square tests.

**TABLE 3:** Sociodemographic characteristics of the study population, by cause of death (respiratory and infectious diseases, including COVID-19, vs. other causes), decedents aged 50+, N = 7,137, (28 European countries. 2018–2022).

Variables	Deceased due to respiratory or infectious diseases, including COVID-19 (%)	Deceased due to other causes (%)	T-test/Chi-square test
N	1,244 (17.4%)	5,893 (82.6%)	
Gender			
Male	689 (55.4%)	3,228 (54.8%)	0.695
Female	555 (44.6%)	2,665 (45.2%)	
Age at death	81.290 years	79.865 years	<0.001
Self-rated health (in the last interview before death)			
Poor/fair	929 (74.7%)	4,253 (72.2%)	0.021
Good	254 (20.4%)	1,228 (20.8%)	
Very good/excellent	61 (4.9%)	412 (7.0%)	
Partnership status			
Has a partner	724 (58.2%)	3,498 (59.4%)	0.450
No partner	520 (41.8%)	2,395 (40.6%)	
Education level			
Low	700 (56.3%)	3,050 (51.8%)	0.008
Medium	401 (32.2%)	2,028 (34.4%)	
High	143 (11.5%)	815 (13.8%)	
Ability to make ends meet			
Easily	221 (17.8%)	1,319 (22.4%)	0.003
Fairly easily	348 (28.0%)	1,585 (26.9%)	
With difficulty	423 (34.0%)	1,920 (32.6%)	
With great difficulty	252 (20.3%)	1,069 (18.1%)	
Geographical area			
Eastern Europe	344 (27.7%)	1,454 (24.7%)	0.011
Northern Europe	140 (11.3%)	817 (13.9%)	
Southern Europe	543 (43.6%)	2,489 (42.2%)	
Western Europe	217 (17.4%)	1,133 (19.2%)	

The table shows the socioeconomic characteristics of the sample, by cause of death. The last column reports p-values from the t-test and chi-square test.

Table 1 shows that the sociodemographic characteristics of older decedents before March 2020 did not differ significantly from those after the outbreak of the pandemic, except for the causes of death and geographical area. A slight difference in age at death is due to the SHARE survey design, with decedents from March 2020 being on average 8 months older than decedents before March 2020.

The share of older decedents due to cardiovascular diseases decreased from 42% to 38% during the pandemic. Decedents due to cancer decreased from 25% to 20% after the outbreak of the pandemic. The shift in the causes of death is likely linked to the insurgence of COVID-19. People with chronic conditions were more at risk of being hospitalised and dying due to COVID-19 [1]. Starting from March 2020, part of the people with chronic diseases such as cardiovascular diseases or cancer, might have contracted COVID-19, resulting in COVID-19 being recorded as their cause of death. According to Eurostat statistics on the causes of death among the population aged over 65, before the pandemic cardiovascular diseases were the cause of death for 32% of decedents while cancer was the leading cause of death for 24% of decedents. After the outbreak of the pandemic, 37% of the decedents aged over 65 were caused by cardiovascular diseases, while 21% by cancer [40]. COVID-19 was already reported as the cause of death for 0.5% of decedents who died before March 2020, which is likely because the circulation of COVID-19 was already known in Europe before March 2020, when the WHO declared COVID-19 to be a pandemic. The geographical distribution of deaths also changed slightly across the two samples. The share of deaths in Eastern Europe increased from 23% to 27% starting from March 2020. Geographical differences might be driven by higher levels of COVID-19 deaths recorded in Eastern European countries. European data on excess mortality show that while Italy and Spain were more affected by the pandemic in the first wave, Eastern European countries such as Bulgaria, Czechia, Poland and Slovakia were more heavily affected in the following waves [41].

Table 2 highlights that decedents who died of COVID-19 and those due to other causes only differed in terms of self-rated health, ability to make ends meet, and geographical area.

Decedents due to COVID-19 were less likely to report poor/fair health (67% among COVID-19 decedents vs. 73% among others). SHARE respondents were asked to self-report their health during the last interview before dying. Such a difference in self-reported health status may appear because people who died due to COVID-19 might have experienced a faster decline in their health due to the contagion from COVID-19. WHO estimates had reported an average of 2–8 weeks between COVID-19 symptoms onset and death [42]. Older decedents due to COVID-19 were also more likely to report difficulties (36%) or great difficulties (24%) in making ends meet, compared to those due to other causes (respectively 32% and 18%). Finally, COVID-19 decedents were more highly concentrated in Eastern Europe compared to those dying of other causes, which confirms the higher level of COVID-19 deaths in these countries compared to the rest of Europe, in line with existing data on excess mortality [41].

Table 3 shows that when dividing the sample between decedents who died due to respiratory and infectious diseases and those who died from other causes, the two groups slightly differed in terms of age, self-rated health, education level, ability to make ends meet and geographical area.

People who died due to respiratory or infectious diseases are slightly older (81 years old on average vs. 79 among those due to other causes) and report a slightly lower health status (75% self-reporting poor or fair health vs. 72% among those due to other causes) in line with existing literature highlighting that more vulnerable people, in older ages or with a poorer health status were more likely to die due to COVID-19 [1, 43]. 56% of decedents due to respiratory or infectious diseases and 52% of decedents due to other causes reported low education. Furthermore, decedents due to respiratory or infectious conditions were 2 percentage points more likely to report making ends meet with difficulty, compared to those who died from other causes. Finally, a higher share of decedents among those who died of respiratory or infectious diseases was in Eastern Europe (28% vs. 25% among decedents for other causes).

**FIGURE 1:**
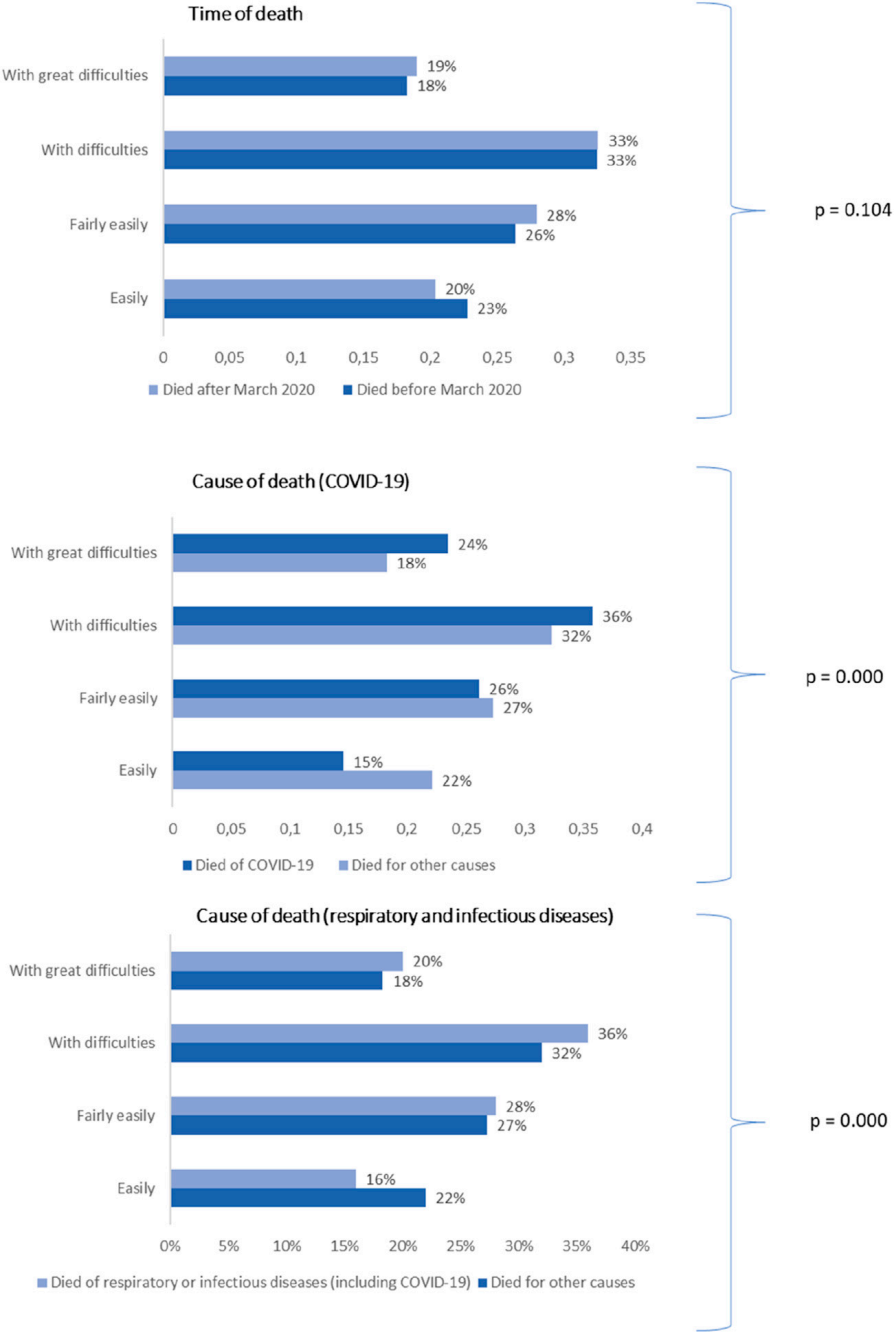
Share of decedents aged 50+ reporting financial difficulties, by time and cause of death (28 European countries. 2018–2022). The figure shows the share of decedents in the sample reporting financial difficulties, by time and cause of death, as well as the p-value from the chi-square tests. Statistical significance: *p < 0.05, **p < 0.01, ***p < 0.001.

Figure 1 presents three graphs: The first illustrates the share of individuals reporting financial difficulties, by time of death. The second shows the share by cause of death, distinguishing between COVID-19 and other causes. The third displays the share of individuals reporting financial difficulties by cause of death, grouping respiratory or infectious diseases (including COVID-19) separately from other causes.

Table 4 reports the results from three logit models. The first model analyses the multivariate association between the time of death and the sociodemographic factors, for all older decedents between 2018 and 2022. The likelihood of dying after March 2020 is higher for decedents who are male (AME = −0.03 p = 0.011, for female), are older (AME = 0.04, p = 0.013 for age group 75–84, AME = 0.05, p = 0.002 for age group 84+), do not have a partner (AME = 0.03, p = 0.031), report financial difficulties (AME = 0.04, p = 0.044 for making ends meet fairly easily, AME = 0.02, p = 0.237 with difficulty, AME = 0.03, p = 0.174 with great difficulty) and live in Eastern Europe (AME = −0.05, p = 0.028 for Northern Europe, AME = −0.05, p = 0.001 for Southern Europe, AME = −0.07, p = 0.000 for Western Europe).

**TABLE 4:** Average Marginal Effects of Logit regression models of the time of death and cause of death on the demographic and socioeconomic characteristics of the decedents aged 50+ from the Survey of Health Ageing and Retirement in Europe, N = 7,137 (28 European countries. 2018–2022).

Variables	Deceased in March 2020 or later	Deceased due to COVID-19	Deceased due to respiratory or infectious disease (incl. COVID-19)
N	7,137	7,137	7,137
Gender (male)			
Female	−0.03*	−0.01	−0.01
	(0.013)	(0.006)	(0.009)
Age at death (50–74)			
75–84	0.04*	0.02*	0.05***
	(0.153)	(0.007)	(0.011)
85+	0.05**	0.02**	0.05***
	(0.158)	(0.008)	(0.012)
Self-rated health (poor/fair)			
Good	−0.01	0.03**	−0.00
	(0.013)	(0.008)	(0.011)
Very good/excellent	−0.03	0.02	−0.03
	(0.018)	(0.139)	(0.017)
Partnership status (has a partner)			
Does not have a partner	0.03*	0.01	−0.00
	(0.013)	(0.008)	(0.010)
Education level (low)			
Medium	0.02	0.01	−0.01
	(0.014)	(0.007)	(0.010)
High	0.03	−0.00	−0.02
	(0.018)	(0.010)	(0.014)
Ability to make ends meet (easily)			
Fairly easily	0.04*	0.01	0.03**
	(0.017)	(0.008)	(0.013)
With difficulty	0.02	0.02*	0.03**
	(0.017)	(0.008)	(0.013)
With great difficulty	0.03	0.02*	0.04**
	(0.020)	(0.010)	(0.015)
Geographical area (Eastern Europe)			
Northern Europe	−0.05*	−0.08***	−0.04**
	(0.020)	(0.009)	(0.015)
Southern Europe	−0.05**	−0.05***	−0.02*
	(0.015)	(0.008)	(0.011)
Western Europe	−0.07***	−0.07***	−0.02
	(0.019)	(0.010)	(0.015)

The table shows average marginal effects and standard errors in parentheses. Statistical significance: *p < 0.05, **p < 0.01, ***p < 0.001. The columns show the results from logit regression models of the three outcome variables.

The second model analyses the multivariate association between the cause of death (COVID-19 vs. other causes) and the sociodemographic factors, for all older decedents between 2018 and 2022. Being older is associated with a higher likelihood of dying of COVID-19 (AME = 0.02, p = 0.020 for age group 75–84, AME = 0.02, p = 0.010 for age group 84+). Having financial difficulties (AME = 0.01, p = 0.190 for making ends meet fairly easily, AME = 0.02, p = 0.038 with difficulty, AME = 0.02, p = 0.021 with great difficulty) and being in Eastern Europe (AME = −0.08, p = 0.000 for Northern Europe, AME = −0.05, p = 0.000 for Southern Europe, AME = −0.07, p = 0.000 for Western Europe) is associated with increased likelihood of dying of COVID-19, in line with results from the previous model. Furthermore, being in good health is slightly associated with a higher likelihood of dying due to COVID-19 (AME = 0.03, p = 0.001, for good health, AME = 0.02, p = 0.278, for very good/excellent health).

The third model introduces a variation in the coding of the cause of death, analysing the multivariate association between the outcome variable on cause of death that has value 1 if the person died due to respiratory or infectious diseases (including COVID-19) and value 0 if the person died from other causes, and the sociodemographic factors. Being older is associated with a higher likelihood of dying due to respiratory or infectious diseases (AME = 0.05, p = 0.000 for age group 75–84, AME = 0.05, p = 0.000, for age group 85+). Having financial difficulties (AME = 0.03, p = 0.013 for making ends meet fairly easily, AME = 0.03, p = 0.016, for with difficulty, AME = 0.04, p = 0.008, for with great difficulty) and living in Eastern Europe (AME = −0.04, p = 0.015 for Northern Europe, AME = −0.02, p = 0.086 for Southern Europe, AME = −0.02, p = 0.133 for Western Europe) is associated with higher likelihood of dying due to respiratory or infectious diseases.

## Discussion

This study uses longitudinal microdata of 7,137 decedents aged over 50 years from 28 European countries, from the Survey on Health, Ageing, and Retirement in Europe (SHARE), to analyse the association of sociodemographic characteristics with time and cause of death.

We found only one cross-country study analysing excess mortality data from 22 European countries in 2020 and 2021 to study the correlation between excess mortality due to the pandemic on mortality rates, and socioeconomic indicators, including life expectancy, *per capita* income and the population’s education level [44]. This study found a higher excess mortality in countries with lower income, lower education and lower life expectancy.

Our analysis found that being male, being older, not having a partner, reporting financial difficulties and living in Eastern European countries were associated with a higher likelihood of dying after March 2020 and of dying due to COVID-19.

### Gender, Age, Partnership Status and Self-Rated Health

Results from the logistic regressions found that males, people of older ages, and those without a partner were more likely to die after March 2020 and to die due to COVID-19, confirming evidence from existing literature [6, 11–20].

Such results might be linked to differences in attitudes towards non-pharmaceutical measures to reduce COVID-19 contagion. Existing literature has highlighted that people of older ages, females, and people with higher education were more likely to comply with non-pharmaceutical measures, such as social distancing and recommendations of regular hands-washing. The opposite holds for people without a partner, people who are employed, and in particular those with regular employment situations (e.g., full time and/or on permanent contracts) [19, 45–48].

Results from the regressions also show that self-reporting good health is positively correlated with the likelihood of dying due to COVID-19 compared to other causes. This result, which seems to contrast the evidence that people with chronic conditions and/or in poorer health were more exposed to the risk of death due to COVID-19 [43], might have several explanations. First of all, the variable on self-reported health builds on information the decedent self-reported in the last interview before death, which might have occurred months or years before their death. Furthermore, people who died of COVID-19 might have experienced a fast decline in their health a few weeks before dying, which could explain our result [42]. Alternatively, the fact that younger people or those in better health were not prioritized by the COVID-19 vaccination campaign in the early phases might explain part of this result [49–51]. It is noteworthy that this result is only significant in the regression using deaths due to COVID-19 as a dependent variable, which could support these explanations.

### Self-Reported Financial Difficulties

The analysis also shows that financial difficulties are associated with an increased likelihood of dying during the pandemic and due to COVID-19. Individuals making ends meet easily were less likely to die during the pandemic compared to those self-reporting financial difficulties. The two analyses using cause of death as an outcome variable confirm such results, showing that financial difficulties were associated with a higher likelihood of dying of COVID-19 or due to respiratory and infectious diseases compared to other causes.

Such results are in line with existing country-specific literature showing that living in more deprived areas or having lower socioeconomic status is associated with higher mortality during the pandemic [12, 21, 23–25, 27–29]. People with lower socioeconomic status might have been more exposed to COVID-19 contagion due to poor housing conditions and less flexible work arrangements, which limited the possibility of social distancing [19, 52]. Furthermore, after contracting COVID-19, people reporting having difficulties making ends meet might have limited access to adequate care. Previous literature had highlighted that financial constraints may limit access to adequate long-term care and end-of-life care, particularly in the last year of life [53–55]. It is important to note that socioeconomic gradients in mortality were present prior to the pandemic and may not have uniformly worsened across all countries.

### Geographical Area

Our logit analysis highlights geographical differences in the exposure to death during the pandemic, with Eastern Europe more exposed to mortality during the pandemic as well as mortality due to COVID-19 compared to other European countries, matching data on excess mortality [41]. This result confirms that higher shares of deaths in Eastern Europe after March 2020 might be driven by higher levels of COVID-19 deaths in these countries. Previous literature suggested that more COVID-19 deaths in Eastern Europe might be linked to a mix of factors, including lower availability of healthcare services, lower health literacy, later implementation of lockdown and non-pharmaceutical measures and lower take up of COVID-19 vaccines compared to other countries [56, 57].

### Practical Implications and Future Research

The findings of this study suggest that the sociodemographic characteristics of older decedents, such as being male, older, not having a partner, having financial difficulties and living in Eastern Europe were associated with an increased likelihood of dying during the pandemic and due to COVID-19. Such findings contribute to a better understanding of the socioeconomic gradients of mortality during the COVID-19 pandemic and might support further work to better understand how public policies can ensure equal access to adequate care for everyone, in any phase of the patient’s pathway. Such findings highlight the relevance of collecting data on the end of life, to contribute to a deeper understanding of the health status, demographic and socioeconomic characteristics, as well as the care experiences of people in the last period of their life. As governments endeavour to improve preparedness and resilience to new shocks to health systems, further research could explore to a greater extent the differences in the experiences of the end of life among people who died before and after the beginning of the pandemic.

### Limitations

This study has limitations due to the nature of SHARE survey data. The analysis relies on proxy respondents and self-reported information from the decedent’s last interview, which may be influenced by personal understanding, recall, and cultural interpretation. Given the cross-national nature of the SHARE survey, this may also result in inconsistencies in the reporting of cause of death across countries, reflecting differences in how proxies interpret and describe health conditions rather than variations in official death certification practices. Additionally, end-of-life interviews require an available proxy, meaning individuals without close relatives may be underrepresented.

While the study accounts for geographical differences, pooling data from 28 countries limits country-specific cultural and socioeconomic insights. Furthermore, this study uses March 2020 as a cut-off point to distinguish between pre- and post-pandemic deaths, based on the WHO’s pandemic declaration. However, this date does not capture the exact onset of COVID-19 circulation in each country, which may vary, potentially introducing minor temporal misclassification. The study also focuses on mortality, preventing an analysis of whether socioeconomic factors influenced exposure to COVID-19, care quality, or both. Due to survey design, post-pandemic decedents are slightly older than pre-pandemic ones, yet the age-stratified analysis reported in Appendix B confirms the robustness of findings.

Alternative approaches, such as Poisson or Cox regression, were not feasible due to the structure of the SHARE survey. The dataset includes only individuals for whom an end-of-life interview was completed, meaning the analysis is limited to decedents, making censoring ambiguous and survival analysis inappropriate. Despite these constraints, SHARE’s rich end-of-life data offer a unique opportunity to examine decedents’ characteristics before and after the pandemic—an opportunity that remains highly valuable.

### Conclusion

Our analysis shows that some sociodemographic characteristics of older decedents are associated with mortality during the pandemic. Being male, older, not having a partner, reporting financial difficulties, and living in Eastern Europe were associated with an increased likelihood of dying during the pandemic and due to COVID-19. As this is an observational study, the findings should be interpreted as associations rather than causal relationships. Furthermore, it is noteworthy that socioeconomic gradients in mortality are longstanding, and it remains challenging to determine whether COVID-19 has intensified or simply reflected these patterns. Yet, this study provides a unique contribution to current literature, by providing individual-level, cross-national evidence covering 28 European countries—in contrast to the predominantly country-specific studies currently available. This paper calls for policies to reduce socioeconomic gradients of mortality and ensure equal access to adequate end-of-life care for everyone with care needs, even during shocks to health systems. Furthermore, such results open the way for further analysis of end-of-life care experiences to better understand the root causes of socioeconomic gradients of mortality of older adults across Europe.

## Data Availability

This paper uses data from SHARE-ERIC (2024). Survey of Health, Ageing and Retirement in Europe (SHARE) Wave 7-9. Release version: 9.0.0. SHARE-ERIC. Data set. Study data already identified are available to the scientific community upon submitting a data request application to the SHARE study.
