# Supplementary material for: Socioeconomic and Demographic Factors Associated With Mortality Before and During the COVID-19 Pandemic: An Analysis of 28 European Countries
Source: Int J Public Health. 2025 Jul 23;70:1608560. doi: 10.3389/ijph.2025.1608560 (PMC12326546; doi:10.3389/ijph.2025.1608560)
Supplement: Supplementary file 1 [file DataSheet1.docx]

**Supplementary material**

**Appendix A. Methods**

**Table A.1. Observations by country**

| **Countries** | **n(2018-2022)** | **n(March 2020- 2022)** |
| --- | --- | --- |
| Austria | 315 | 168 |
| Belgium | 322 | 159 |
| Bulgaria | 154 | 72 |
| Croatia | 309 | 169 |
| Cyprus | 65 | 25 |
| Czech Republic | 438 | 225 |
| Denmark | 198 | 76 |
| Estonia | 686 | 328 |
| Finland | 43 | 27 |
| France | 188 | 79 |
| Germany | 209 | 104 |
| Greece | 405 | 187 |
| Hungary | 290 | 160 |
| Israel | 186 | 91 |
| Italy | 474 | 262 |
| Latvia | 144 | 102 |
| Lithuania | 185 | 87 |
| Malta | 65 | 35 |
| Netherlands | 133 | 51 |
| Poland | 568 | 334 |
| Portugal | 140 | 60 |
| Romania | 287 | 162 |
| Slovenia | 434 | 224 |
| Spain | 333 | 162 |
| Sweden | 322 | 147 |
| Switzerland | 133 | 56 |
| Luxembourg | 39 | 19 |
| Slovakia | 61 | 21 |
|  |  |  |
| Total | 7137 | 3592 |

*The table reports the number of observations by country for the whole sample (decedents who died between 2018 and 2022) and for decedents who died during the pandemic (from March 2020 to 2022).*

**Table A.2. End-of-life interview questions from Survey on Health Ageing and Retirement in Europe used to build the outcome variables**

| **Questions asked to proxy respondent to identify the decedent’s month and year of death** | |
| --- | --- |
| XT008_MonthDied (Month of decease) | We would like to know more about the circumstances of [Name of the deceased] 's death. In what **month** and year did [he/ she] pass  away?  1. January  2. February  3. March  4. April  5. May  6. June  7. July  8. August  9. September  10. October  11. November  12. December |
| XT009_YearDied (Year of decease) | In what month and **year** did [he/ she] pass away?  1. 2006  2. 2007  3. 2008  4. 2009  5. 2010  6. 2011  7. 2012  8. 2013  9. 2014  10. 2015  11. 2016  12. 2017  13. 2018  14. 2019  15. 2020  16. 2021  17. 2022  18. 2023  19. 2024 |
| **Question asked to proxy respondent to identify the decedent’s main cause of death** | |
| XT011_CauseDeath (The main cause of death) | What was the main cause of [his/ her] death?  1. Cancer  2. A heart attack  3. A stroke  4. Other cardiovascular related illness such as heart failure, arrhythmia  5. Respiratory disease  6. Disease of the digestive system such as gastrointestinal ulcer, inflammatory bowel disease  7. Severe infectious disease such as pneumonia, septicaemia or flu  8. Accident or suicide  9. Covid-19 or related complications  97. Other (Please specify) |

**Appendix B. Stratified Logit regression**

**Table B.1. Average Marginal Effects of age-stratified Logit regression of the time and cause of death on sociodemographic characteristics of decedents aged between 50 and 79, N=3,069 (28 European countries. 2018-2022)**

| **Variables** | **Deceased in March 2020 or later** | **Deceased due to COVID-19** | **Deceased due to respiratory or infectious disease (incl. COVID-19)** |
| --- | --- | --- | --- |
| **N** | 3,069 | 3,069 | 3,069 |
| **Gender (male)** |  |  |  |
| Female | -0.02 | 0.00 | 0.00 |
|  | (0.019) | (0.009) | (0.014) |
|  |  |  |  |
| **Self-rated health (poor/fair)** |  |  |  |
| Good | 0.00 | 0.02 | -0.00 |
|  | (0.021) | (0.011) | (0.015) |
| Very good / excellent | -0.03 | 0.00 | -0.06* |
|  | (0.032) | (0.015) | (0.019) |
|  |  |  |  |
| **Partnership status (has a partner)** |  |  |  |
| Does not have a partner | 0.03 | 0.01 | -0.00 |
|  | (0.020) | (0.010) | (0.014) |
|  |  |  |  |
| **Education level (low)** |  |  |  |
| Medium | 0.04* | 0.00 | -0.01 |
|  | (0.019) | (0.009) | (0.014) |
| High | 0.03 | -0.02* | -0.03 |
|  | (0.029) | (0.012) | (0.020) |
|  |  |  |  |
| **Ability to make ends meet (easily)** |  |  |  |
| Fairly easily | 0.05 | 0.00 | 0.04* |
|  | (0.027) | (0.014) | (0.019) |
| With difficulty | 0.01 | 0.01 | 0.04* |
|  | (0.027) | (0.014) | (0.018) |
| With great difficulty | 0.03 | 0.01 | 0.04* |
|  | (0.031) | (0.015) | (0.021) |
|  |  |  |  |
| **Geographical area (Eastern Europe)** |  |  |  |
| Northern Europe | -0.03 | -0.07*** | -0.04* |
|  | (0.030) | (0.013) | (0.021) |
| Southern Europe | -0.06** | -0.05*** | -0.04* |
|  | (0.022) | (0.011) | (0.015) |
| Western Europe | -0.06** | -0.07*** | -0.02 |
|  | (0.028) | (0.012) | (0.021) |

*The table shows average marginal effects and standard errors in parentheses. Statistical significance: * p < 0.05, ** p < 0.01, *** p < 0.001. The columns show the results from age-stratified logit regression models of the three outcome variables on the covariates.*

**Table B.2. Average Marginal Effects of age-stratified Logit regression of the time and cause of death on the sociodemographic characteristics of decedents aged 80 or older, N=4,068 (28 European countries. 2018-2022)**

| **Variables** | **Deceased in March 2020 or later** | **Deceased due to COVID-19** | **Deceased due to respiratory or infectious disease (incl. COVID-19)** |
| --- | --- | --- | --- |
| **N** | 4,068 | 4,068 | 4,068 |
| **Gender (male)** |  |  |  |
| Female | -0.04* | -0.00 | -0.03* |
|  | (0.018) | (0.009) | (0.012) |
|  |  |  |  |
| **Self-rated health (poor/fair)** |  |  |  |
| Good | -0.02 | 0.03** | 0.02 |
|  | (0.020) | (0.011) | (0.014) |
| Very good / excellent | -0.03 | 0.04 | 0.03 |
|  | (0.018) | (0.025) | (0.028) |
|  |  |  |  |
| **Partnership status (has a partner)** |  |  |  |
| Does not have a partner | 0.03 | 0.01 | 0.01 |
|  | (0.017) | (0.009) | (0.012) |
|  |  |  |  |
| **Education level (low)** |  |  |  |
| Medium | 0.01 | 0.00 | -0.01 |
|  | (0.018) | (0.010) | (0.013) |
| High | 0.02 | 0.01 | 0.01 |
|  | (0.024) | (0.015) | (0.017) |
|  |  |  |  |
| **Ability to make ends meet (easily)** |  |  |  |
| Fairly easily | 0.03 | 0.02 | 0.04** |
|  | (0.022) | (0.011) | (0.015) |
| With difficulty | 0.02 | 0.03* | 0.04** |
|  | (0.023) | (0.012) | (0.015) |
| With great difficulty | 0.02 | 0.04* | 0.04* |
|  | (0.027) | (0.014) | (0.018) |
|  |  |  |  |
| **Geographical area (Eastern Europe)** |  |  |  |
| Northern Europe | -0.05 | -0.08*** | -0.05** |
|  | (0.028) | (0.014) | (0.018) |
| Southern Europe | -0.04 | -0.05*** | -0.02 |
|  | (0.020) | (0.013) | (0.015) |
| Western Europe | -0.07** | -0.06*** | -0.03 |
|  | (0.026) | (0.015) | (0.018) |

*The table shows average marginal effects and standard errors in parentheses. Statistical significance: * p < 0.05, ** p < 0.01, *** p < 0.001. The columns show the results from age-stratified logit regression models of the three outcome variables on the covariates.*
